# Traits-Based Integration of Multi-Species Inoculants Facilitates Shifts of Indigenous Soil Bacterial Community

**DOI:** 10.3389/fmicb.2018.01692

**Published:** 2018-07-26

**Authors:** Jingjing Wang, Qingqing Li, Song Xu, Wei Zhao, Yu Lei, Chunhui Song, Zhiyong Huang

**Affiliations:** ^1^Key Laboratory of Western China’s Mineral Resources of Gansu Province, School of Earth Sciences, University of Lanzhou, Lanzhou, China; ^2^Tianjin Key Laboratory for Industrial Biosystems and Bioprocessing Engineering, Tianjin Institute of Industrial Biotechnology, Chinese Academy of Sciences, Tianjin, China; ^3^Core Facility, Tianjin Institute of Industrial Biotechnology, Chinese Academy of Sciences, Tianjin, China

**Keywords:** microbial co-inoculants, integration, bacterial community structure and function, soil properties, soil enzyme, cucumber

## Abstract

Microbial co-inoculation is considered to be an innovative approach and had been applied worldwide. However, the underlying mechanisms of microbial co-inoculants constructions, especially the trait-based combination of distinctly different microbial species remains poorly understood. In this study, we constructed two microbial co-inoculants with the same three strains with emphasis on the microbial, soil and plant traits. Microbial co-inoculants 1 (M1) were constructed according to soil fertility, microbial activity and cucumber nutrient requirement with a 2:1:2 ratio (*Ensifer* sp. NYM3, *Acinetobacter* sp. P16 and *Flavobacterium* sp. KYM3), while microbial co-inoculants 2 (M2) were constructed according to soil fertility and cucumber nutrient requirement with a 1:10:1 ratio without considering the difference in the nutrient supply capability of microbial species. The results showed that M1 and M2 both obviously increased cucumber yields. The M1 had significant highest pH value, total nitrogen (TN) and invertase activity (IA). The M2 had significant highest available phosphate (AP), NO_3_-N, urea activity (UA), and alkaline phosphatase activity (APA). Gammaproteobacteria, Acidobacteria, Nitrospirae, and Armatimonadetes were significantly increased, while Actinobacteria and Firmicutes were significantly decreased by microbial co-inoculations (M1 and M2). The bacterial lineages enriched in M1 were Gammaproteobacteria and TM7. Acidobacteria, Bacteroidetes, and Deltaproteobacteria were enriched in M2. Principal coordinate analysis (PCoA) analysis showed that the bacterial communities were strongly separated by the different microbial inoculation treatments. The functional groups of intracellular_parasites were highest in M1. The functional groups of phototrophy, photoautotrophy, nitrification, fermentation, cyanobacteria, oxygenic_photoautotrophy, chitinolysis and animal_parasites_or_symbionts were highest in M2. Based on correlation analysis, it inferred that the M1 and M2 might promote cucumber yields by mediating bacterial community structure and function about nitrogen fixing and urea-N hydrolysis, respectively. Collectively, these results revealed that microbial co-inoculants had positive effects on cucumber yields. Trait-based integration of different microbial species had significant effects on soil properties and bacterial communities. It indicated that microbial activity should be considered in the construction of microbial co-inoculants. This will expand our knowledge in bacteria interaction, deepen understanding of microbial inoculants in improving plant performance, and will guide microbial fertilizer formulation and application in future.

## Introduction

Microbial inoculants, especially plant growth-promoting bacteria (PGPB) inoculants, are an alternative method of increasing crop productivity that can reduce the use of pesticides and/or chemical fertilizers (Adesemoye et al., 2009; Beneduzi et al., 2012; Baris et al., 2014; Pereg and McMillan, 2015; Baez-Rogelio et al., 2017). PGPB can promote plant growth through providing nutrient, producing phyto-hormone, defending against pathogens, alleviating stress and so on (Saleem et al., 2007; Bhattacharyya and Jha, 2012; Ambrosini et al., 2016). In order to enhance the reliability and efficacy of microbial inoculants in agriculture, combining these PGPB by co-inoculation had been applied worldwide for more than a decade. As co-inoculation would mimic the natural situation more closely and allow the combination of various mechanisms without the need for genetic engineering (Madhaiyan et al., 2010; Rodrigues et al., 2013). Microbial co-inoculants construction includes species selection and species integration. Microbial co-inoculants were usually composed of two or more compatible strains with different functions. The common selections were nitrogen fixing bacteria (*Azospirillum* sp. or Rhizobia) and biocontrol agents (*Pseudomonas* sp. or *Bacillus* sp.) (Figueiredo et al., 2008; Atieno et al., 2012b). Species integrations were essentially the ratios of microbial species in co-inoculants. The ratios of microbial species in co-inoculants were mostly 1:1 (Maheshwari et al., 2010; Yegorenkova et al., 2016). Saleem et al. (2016) revealed that species richness had positive effects on efficiency and stabilize of microbial co-inoculants and different ratios of a specie had different effects on co-inoculants efficiency. However, Paredes et al. (2018) demonstrated that the effects of microbial co-inoculants on plant physiology were mostly additive and independent of bacterial abundances. Therefore, multi-species with different ratios in inoculants should be paid more attentions.

After releasing in field, microbial inoculants efficiency is not only associated with its beneficial features, but also with the complex network of interactions occurring in the soil ecosystem (Johnke et al., 2014; Ambrosini et al., 2016; Shi et al., 2016). The knowledge of the effects of microbial inoculants on microbial community has been of great interest since changes in the structure of the indigenous microbial communities might affect the function of microbes plays in soil (Berendsen et al., 2012; Mueller and Sachs, 2015). Schwieger and Tebbe (2000) firstly reported the effect of field inoculation with *Sinorhizobium meliloti* L33 on bacterial communities in rhizospheres of *Medicago sativa* by SSCP (single-strand conformation polymorphism). The results showed that *Sinorhizobium meliloti* L33 inoculation decreased γ-proteobacteria and increased α-proteobacteria. Then many investigates indicated that application of microbial inoculants, such as rhizobia, *Azospirillum*, AMF, biocontrol agents and co-inoculation, could influence the resident microbial communities and commonly increased soil microbial diversity by various culture-independent methods, such as DGGE (denaturing-gradient gel electrophoresis), PLFA (phospholipid fatty acid) and the latest high-throughput sequencing (Barriuso et al., 2008; Felici et al., 2008; Trabelsi and Mhamdi, 2013; Pang et al., 2017; Rodriguez-Caballero et al., 2017). The positive effects of microbial inoculants on microbial diversity had also been demonstrated in many ecosystems (Xi et al., 2015; Zhang et al., 2017). However, the effect of different ratios of multi-species in inoculants on soil indigenous microbial community is rarely explored.

In this study, we constructed two microbial co-inoculants according to soil fertility, microbial activity and cucumber nutrient requirement. The two microbial co-inoculants had the same three strains but with different ratios. The present study aims to determine whether the two different ratios (2:1:2 vs. 1:10:1) of the integrated multi-species, comprising of three selected bio-inoculants, viz. *Ensifer* sp. NYM3 (Alphaproteobacteria), *Acinetobacter* sp. P16 (Gammaproteobacteria), and *Flavobacterium* sp. KYM3 (Bacteroidetes), have the same effects on cucumber yield, soil properties, especially on soil indigenous bacterial community in the field experiment. The ultimate goal is to choose the better construction of microbial co-inoculants. This work will provide valuable suggestion for microbial fertilizer formulation and application in future.

## Materials and Methods

### Field Site and Experiment Description

The experiment was performed in a cucumber (Jing Pin Xin Xiu, Dalian Shuangfeng seeds Co. Ltd., China) field in Anshan, Liaoning Province, China (41°00′ N, 123°00′ E). This region has a warm temperate continental monsoon climate with an average annual temperature of 7.5°C and a mean annual precipitation of 707 mm. The soil type is classified as brown earth. The soil had the following physio-chemical properties: pH 6.8, organic carbon 23.5 g/kg, total nitrogen (TN) 1.42 g/kg, total phosphorus 0.107 g/kg, total potassium 3.1 g/kg, available nitrogen 147 mg/kg, available phosphorus 6 mg/kg, available potassium 376 mg/kg.

The *Ensifer* sp. NYM3, the other name is *Sinorhizobium* sp. NYM3 (KY203668), *Acinetobacter* sp. P16 (KY203666), and *Flavobacterium* sp. KYM3 (KY203667) were previously isolated from Mount Huang in Anhui Province, China by Ashby culture medium, inorganic phosphate culture medium and K-releasing culture medium, respectively (Xu et al., 2015; Yin et al., 2015). The nitrogen-fixing activity of *Ensifer* sp. NYM3 was 921 nmol C_2_H_2_/h⋅mg protein. The phosphate solubilizing ability of *Acinetobacter* sp. P16 was 120 mg/L. The potassium solubilizing ability of *Flavobacterium* sp. KYM3 was 68 mg/L. Previous pot experiment showed that all the three strains had positive effects on cucumber. Previous antagonistic experiments showed that there was no antagonism among the three strains.

Microbial co-inoculants 2 (M2) were constructed according to soil fertility and cucumber nutrient requirement without considering the difference in the nutrient supply capability of microbial species. Soil nitrogen and potassium were about 10 times as much as soil phosphorus. The amount of nitrogen, phosphorus and potassium absorbed by 100 kg cucumber yields was about 0.4 kg, 0.4 kg, and 0.5 kg, respectively. Therefore, the ratio of *Ensifer* sp. NYM3, *Acinetobacter* sp. P16 and *Flavobacterium* sp. KYM3 in M2 was set as 1:10:1.

Microbial co-inoculants 1 (M1) were constructed according to soil fertility, microbial activity and cucumber nutrient requirement. We calculated the ratio of *Acinetobacter* sp. P16 and *Flavobacterium* sp. KYM3 as follows:

$$\left(\frac{\text{AP}}{\text{TP}}+120\text{X}\right):\left(\frac{\text{AK}}{\text{TK}}+68\text{Y}\right)=0.4:0.5$$

Where AP is the available phosphorus, TP is the total phosphorus, AP/TP is the soil phosphate solubilizing ability, 120 is the phosphate solubilizing ability of *Acinetobacter* sp. P16, X is the quantity of *Acinetobacter* sp. P16, AK is the available potassium, TK is the total potassium, AK/TK is the soil potassium solubilizing ability, 68 is the potassium solubilizing ability of *Flavobacterium* sp. KYM3, X is the quantity of *Flavobacterium* sp. KYM3, 0.4:0.5 was the ratio of phosphorus and potassium absorbed by 100 kg cucumber yields. Through this formula, we get the ratio of *Acinetobacter* sp. P16 and *Flavobacterium* sp. KYM3 to about 1:2. Because nitrogen is the largest nutrient element of plant demand, we set the ratio of *Ensifer* sp. NYM3, *Acinetobacter* sp. P16 and *Flavobacterium* sp. KYM3 in M1 as 2:1:2.

The fertilization experiments were established in 2014 and were designed in a randomized complete block with three replicates, each measuring 40 m^2^. The treatments were the non-inoculated (CK), microbial co-inoculants 1 (M1), microbial co-inoculants 2 (M2). Each plot received 4.08 kg organic and chemical compound fertilizer [lignite 70%, KH_2_PO_4_ 5.51%, (NH_4_)_2_PO_4_ 24.49%] at three different growth stages (base fertilizer, flowering period, and fruiting period), respectively. Microbial co-inoculants (1.5 × 10^14^cfu/ha) were applied with organic and chemical compound fertilizer and were only added in plots treated with M1 and M2.

The yields of cucumber from the entire plot were weighed and recorded during harvest period. Surface soils (0–20 cm) near cucumber roots were randomly sampled from 10 points in each treatment plot with a 2.5 cm diameter auger after cucumber harvest on July 2014, and thoroughly mixed into one composite sample. A total of nine composite samples (3 treatments × 3 replicates = 9 samples) were collected. Each soil sample was pooled in a sterile plastic bag, and transported to the laboratory on ice. The samples were sieved (2 mm) and then stored at 4°C (for soil characterization) or −80°C (for soil DNA isolation) until analysis.

### Soil Characteristics

The soil pH, organic matter (OM), total nitrogen (TN), total phosphorus (TP), total potassium (TK), available nitrogen (AN), available phosphorus (AP), available potassium (AK), nitrate nitrogen (NO_3_-N), invertase activity (IA), urease activity (UA), alkaline phosphatase activity (APA), and catalase activity (CA) were determined using commercial chemical assay kits (Suzhou Comin Biotechnology Co., Ltd., China), respectively, following the manufacturer’s instructions.

### Nucleic Acid Isolation and Amplification of 16S rRNA Genes

To analyze the bacterial community richness and composition, soil DNA was isolated from the three treatments by the PowerSoil DNA isolation kit (MoBio Laboratories, Inc., Carlsbad, CA, United States). The genomic DNA were quantified with a NanoDrop ND-2000 spectrophotometer (NanoDrop Technologies, Wilmington, DE, United States) and stored at −80°C until amplification.

Briefly, the bacterial hypervariable domain V4 was amplified with region-specific primers (515F: 5′-GTGCCAGCMGCCGCGGTAA-3′ and 806R: 5′-GGACTACHVGGGTWTCTAAT-3′) that included the Illumina flowcell adapter sequences (Caporaso et al., 2012). The reverse amplification primers contained a 10-base barcode sequence. The purified PCR products were normalized in equimolar amounts in a single tube for MiSeq 2000 sequence runs (Illumina, San Diego, CA, United States), producing bidirectional reads.

### Bioinformatic Analysis of 16S rRNA Gene Sequences

The 16S rRNA gene sequences were processed and analyzed by QIIME 1.9 (Caporaso et al., 2012). Initially, sequences shorter than 200 bp, containing unresolved nucleotides, exhibiting an average quality score lower than 25, harbor mismatches longer than 3 bp in the forward primer, or possessing homopolymers longer than 6 bp were removed with *split_libraries.py*. Chimeric sequences were removed using UCHIME with Ribosomal Database Project (RDP) as reference dataset (trainset10_082014_rmdup.fasta). Operational taxonomic unit (OTU) determination was performed at a genetic divergence of 3% (species level) with *pick_open_reference_otus.py*. Singletons, chloroplasts, unclassified OTUs and extrinsic domain OTUs were removed by employing *filter_otu_table.py*. We used a randomly selected subset of 6,500 sequences per sample to calculate the diversities and distances between samples using *core_diversity_analyses.py*. Differentially abundant OTUs between treatment groups were identified using linear discriminant analysis (LDA) effect size (LEfSe) analysis (Segata et al., 2011). Microbial community function were predicted by Functional Annotation of Prokaryotic Taxa (FAPROTAX) using *python collapse_table.py* (Louca et al., 2016).

### Accession Numbers

The 16S rRNA gene sequences were deposited in the National Center for Biotechnology Information (NCBI) Sequence Read Archive (SRA) under accession number SRP127639.

### Statistical Analysis

All statistical analyses were performed using R (version 3.1.1) and IBM SPSS Statistics 19 (SPSS Inc.). The effects of the different treatments on the cucumber yields, soil physicochemical properties, soil enzymes activity, α-diversity indices and relative abundance of abundant taxa were tested using one-way ANOVA followed by Tukey’s *post hoc* test. Principal coordinate analysis (PCoA) and analysis of similarity (ANOSIM) based on weighted UniFrac distance were performed to evaluate the overall differences in the bacterial community. Permutational multivariate analysis of variance (PERMANOVA) with ADONIS function was conducted to quantitatively evaluate the contribution of different treatments to the variations of the bacterial community (Xiong et al., 2015). These analyses were performed in R with the ‘vegan’ package. Spearman’s rank correlations between abundant taxa, cucumber yield, and soil properties were calculated using IBM SPSS Statistics 19 (SPSS Inc.). A heatmap that illustrates the Functional Annotation of Prokaryotic Taxa (FAPROTAX) data underlying the clustering patterns was generated using a software package of HemI (Heatmap Illustrator, version 1.0.1) (Deng et al., 2014).

## Results and Discussion

### Cucumber Yields

**FIGURE 1:**
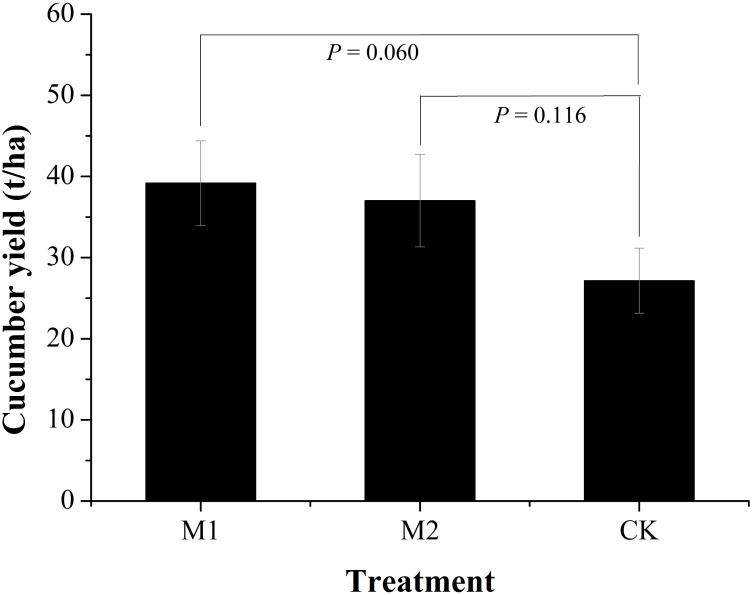
Cucumber yields under different treatments. M1, microbial co-inoculants 1; M2, microbial co-inoculants 2; CK, non-inoculated.

Microbial co-inoculation treatments (M1 and M2) obviously increased cucumber yields compared to the non-inoculated treatment (CK) (**Figure 1**). The M1 increased 44.23% while M2 increased 36.32% of cucumber yields. There was no obvious difference in cucumber yields between the microbial co-inoculation treatments. The M1 treatment resulted in the higher yield of cucumber (39.16 t/ha) that that of M2 (37.02 t/ha).

### Soil Physicochemical Characteristics and Enzymatic Activity

The soil pH, TN, AK, NO_3_-N, IA, UA and APA contents differed significantly between the treatments (*P* < 0.05) (**Table 1**). The soil pH, TN and IA were significantly higher in M1 than that in other treatments. The UA and PA was significantly higher in M2 than in other treatments. M1 had significantly higher TN than M2, and M2 had significantly higher TN than CK. M2 had significantly higher UA than M1, and M1 had significantly higher UA than CK. M2 had significantly higher AK and NO_3_-N than that in M1.

**Table 1:** Physicochemical and biological characteristics of cucumber soil under different treatments.

Treatment	M1	M2	CK
pH	6.82 ± 0.23a	6.34 ± 0.24b	6.24 ± 0.07b
OM (g kg^−1^)	11.47 ± 1.04a	12.63 ± 1.01a	12.04 ± 1.54a
TN (g kg^−1^)	1.35 ± 0.01a	1.17 ± 0.05b	1.01 ± 0.02c
TP (g kg^−1^)	0.27 ± 0.02a	0.28 ± 0.02a	0.27 ± 0.00a
TK (g kg^−1^)	2.59 ± 0.16a	2.45 ± 0.08a	2.25 ± 0.18a
AN (mg kg^−1^)	77.39 ± 9.58a	69.11 ± 2.28a	67.22 ± 6.806a
AP (mg kg^−1^)	166.52 ± 12.92a	144.08 ± 14.76a	154.56 ± 22.71a
AK (mg kg^−1^)	47.7 ± 2.84b	64.92 ± 4.81a	62.88 ± 11.61ab
NO_3_-N (mg kg^−1^)	8.17 ± 0.82b	17.84 ± 5.43a	11.70 ± 2.18ab
IA (mg g^−1^ d^−1^)	21.76 ± 0.63a	11.20 ± 1.78b	12.11 ± 2.48b
UA (mg g^−1^ d^−1^)	1.08 ± 0.03b	1.15 ± 0.03a	0.89 ± 0.02c
APA (mg g^−1^ d^−1^)	0.26 ± 0.01b	0.47 ± 0.02a	0.27 ± 0.03b
CA (mg g^−1^ 20 min^−1^)	0.01 ± 0.00a	0.01 ± 0.00a	0.01 ± 0.00a

*OM, organic matter; TN, total nitrogen; TP, total phosphorus; TK, total potassium; AN, available nitrogen; AP, available phosphorus; AK, available potassium; NO_3_-N, nitrate nitrogen; IA, invertase activity; UA, urease activity; APA, alkaline phosphatase activity; CA, catalase activity. M1, microbial co-inoculants 1; M2, microbial co-inoculants 2; CK, non-inoculated. Values (means ± SD, n = 3) within the same row followed by different letters are significantly different at P < 0.05 according to Tukey’s post hoc test.*

### Bacterial Community Structure Response to Microbial Inoculation

**FIGURE 2:**
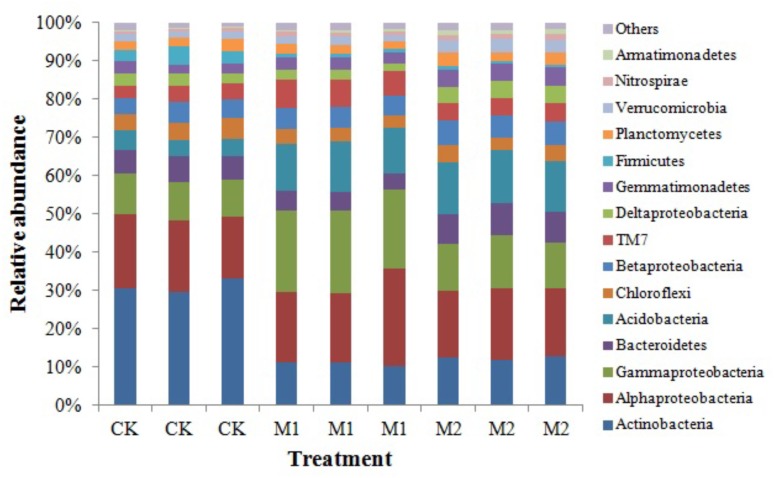
Relative abundances of the dominant bacterial phyla (proteobacterial classes) under different treatments. M1, microbial co-inoculants 1; M2, microbial co-inoculants 2; CK, non-inoculated.

Across all samples, we obtained a total of 133,394 high-quality sequences and 6534–31354 sequences per sample (mean = 14,821). These high-quality sequences were clustered into 13, 226 OTUs at 97% sequence similarity, with 1521–2571 OTUs per sample. After rarefied to 6,500 sequences per sample, Actinobacteria, Alphaproteobacteria, Gammaproteobacteria, Bacteroidetes, Acidobacteria, Chloroflexi, Betaproteobacteria, TM7, Deltaproteobacteria, Gemmatimonadetes, Firmicutes, Planctomycetes, Verrucomicrobia, Nitrospirae, and Armatimonadetes were the dominant phyla (proteobacterial classes) (>1%) across all treatments. These dominant phyla were accounting for more than 97% of the bacterial sequences from each soil sample (**Figure 2**). The phyla (proteobacterial classes) Actinobacteria, Gammaproteobacteria, Bacteroidetes, Acidobacteria, Chloroflexi, Betaproteobacteria, TM7, Deltaproteobacteria, Gemmatimonadetes, Firmicutes, Verrucomicrobia, Nitrospirae, and Armatimonadetes varied significantly (*P* < 0.05) between the different treatments (**Table 2**). Gammaproteobacteria, Acidobacteria, Nitrospirae, and Armatimonadetes were significantly increased by microbial co-inoculation (M1 and M2) (*P* < 0.05). Conversely, Actinobacteria and Firmicutes showed the opposite pattern. M1 had highest TM7 and lowest Chloroflexi (*P* < 0.05). M2 had highest Gemmatimonadetes and Verrucomicrobia (*P* < 0.05). M2 had significant higher Betaproteobacteria than CK (*P* < 0.05). Bacteroidetes and Deltaproteobacteria were significant highest in M2 and lowest in M1 (*P* < 0.05). Additionally, no significant difference in Alphaproteobacteria and Planctomycetes was observed between different treatments.

**Table 2:** Statistically significant differences in the dominant bacterial phyla (proteobacterial classes) under different treatments.

	M1	M2	CK
Actinobacteria	10.92 ± 0.55b	12.46 ± 0.48b	31.18 ± 1.72a
Alphaproteobacteria	20.63 ± 4.22a	17.94 ± 0.62a	17.97 ± 1.58a
Gammaproteobacteria	21.22 ± 0.44a	12.71 ± 1.15b	10.21 ± 0.50c
Bacteroidetes	4.75 ± 0.57c	8.10 ± 0.36a	6.31 ± 0.45b
Acidobacteria	12.37 ± 0.67a	13.55 ± 0.30a	4.56 ± 0.39b
Chloroflexi	3.71 ± 0.38b	3.91 ± 0.62a	4.75 ± 0.56a
Betaproteobacteria	5.30 ± 0.20ab	6.14 ± 0.13a	4.90 ± 0.69b
TM7	7.03 ± 0.36a	4.57 ± 0.40b	3.82 ± 0.47b
Deltaproteobacteria	2.28 ± 0.31c	4.50 ± 0.20a	3.05 ± 0.29b
Gemmatimonadetes	3.09 ± 0.38b	4.57 ± 0.10a	2.73 ± 0.41b
Firmicutes	1.02 ± 0.01b	0.76 ± 0.24b	3.67 ± 0.92a
Planctomycetes	2.25 ± 0.29a	3.09 ± 0.47a	2.56 ± 0.57a
Verrucomicrobia	1.91 ± 0.32b	3.26 ± 0.08a	1.78 ± 0.24b
Nitrospirae	1.09 ± 0.07a	1.34 ± 0.14a	0.74 ± 0.09b
Armatimonadetes	0.51 ± 0.07b	1.19 ± 0.07a	0.34 ± 0.05c
Others	1.91 ± 0.13a	1.91 ± 0.12a	1.42 ± 0.40a

*M1, microbial co-inoculants 1; M2, microbial co-inoculants 2; CK, non-inoculated. Values (means ± SD, n = 3) within the same row followed by different letters are significantly different at P < 0.05 according to Tukey’s post hoc test.*

The high throughout sequencing results also showed that the relative abundances of *Flavobacterium* was significant higher than that of Rhizobiaceae_other and *Acinetobacter* sp. in M1. The ratio of Rhizobiaceae_other, *Acinetobacter* sp. and *Flavobacterium* sp. in M1 was about 2:1:14. The relative abundances of *Flavobacterium* sp. was significant higher than that of Rhizobiaceae_other in M2. The relative abundances of Rhizobiaceae_other was significant higher than that of *Acinetobacter* sp. in M2. The ratio of Rhizobiaceae_other, *Acinetobacter* sp. and *Flavobacterium* sp. in M2 was about 12:1:24. The relative abundances of Rhizobiaceae_other, *Acinetobacter* sp. and *Flavobacterium* sp. had no significant difference in CK. The ratio of Rhizobiaceae_other, *Acinetobacter* sp. and *Flavobacterium* sp. in CK was about 14: 2: 10 (Supplementary Figure S1).

The bacterial α-diversity in the individual samples under the different treatments was calculated. The number of OTUs, phylogenetic diversity, and the Shannon–Weaver index had no significant changes among the three different treatments (Supplementary Figure S2). It was obvious that the bacterial α-diversity (the number of OTUs, phylogenetic diversity, and the Shannon–Weaver index) in M2 were generally higher than the other treatments.

**FIGURE 3:**
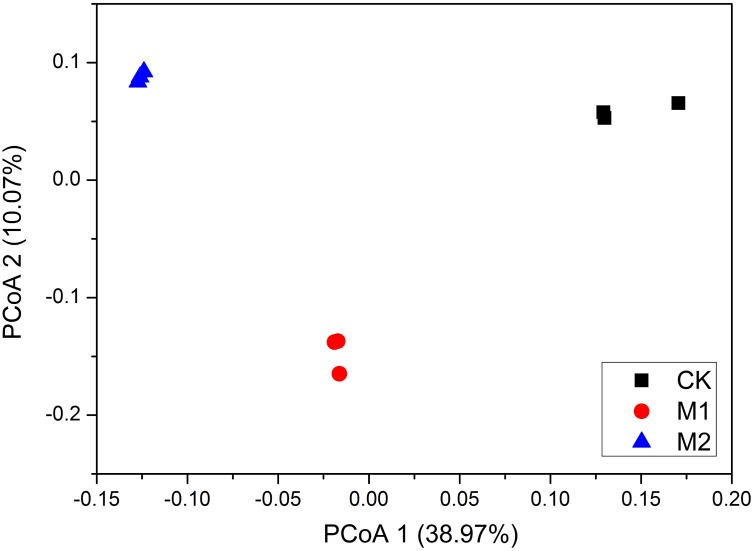
Principal coordinate analysis (PCoA) of weighted UniFrac distances of the bacterial communities under different treatments. M1, microbial co-inoculants 1; M2, microbial co-inoculants 2; CK, non-inoculated.

The PCoA revealed that the bacterial communities were obviously separated by the three treatments (**Figure 3**). ANOSIM analysis showed that microbial co-inoculation treatments (global R = 1, *P* = 0.003) were significant factors in shaping the bacterial community composition. Accordingly, ADONIS analysis showed that microbial co-incubation treatment contributed 82.3% (*P* = 0.01) of community variance. Collectively, these results demonstrated that the composition and structure of microbial communities were predominantly affected by the different microbial co-inoculation treatments.

### Bacterial Groups With Statistical Differences

**FIGURE 4:**
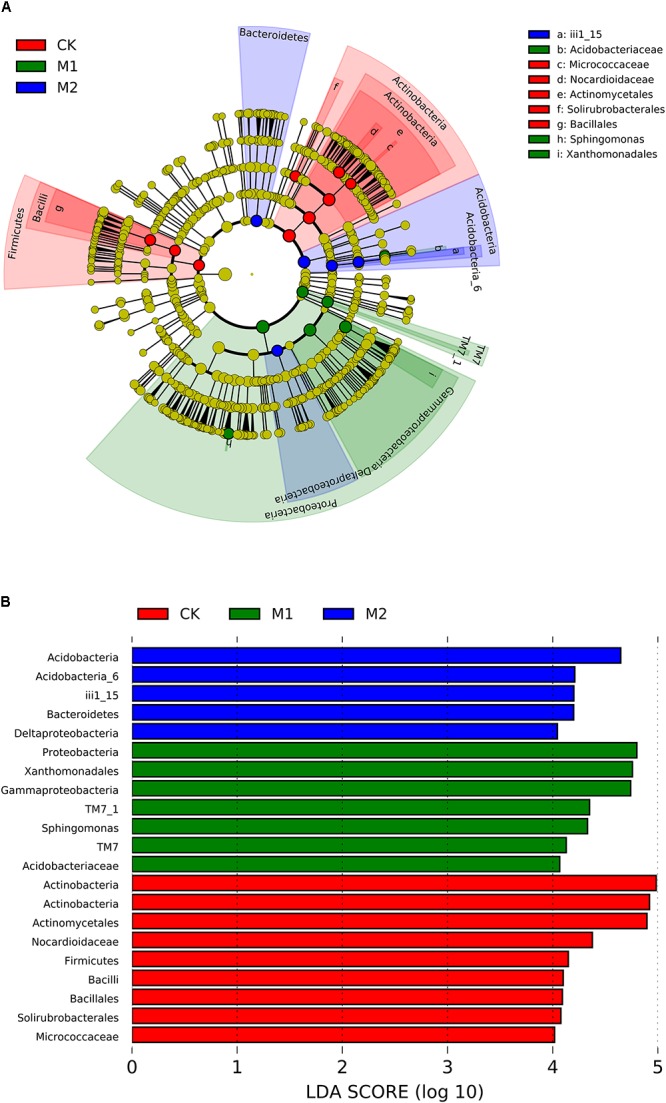
Cladogram **(A)** and LDA score **(B)** of LEfSe analysis of bacterial community among CK (Red), M1 (green) and M2 (blue) treatments. Only the taxa with meeting a significant LDA threshold value of >4 were shown. M1, microbial co-inoculants 1; M2, microbial co-inoculants 2; CK, non-inoculated.

To further investigate which bacterial taxa were distinct among the groups, LEfSe analysis was applied. A total of 153 bacterial groups were distinct to at least one treatment using the default logarithmic (LDA) value of 2 (Supplementary Figure S3). Cladograms show taxa with LDA value of 4 for clarity (**Figure 4**). The bacterial lineages enriched in M1 were Proteobacteria (the phylum and its class Gammaproteobacteria, and its orders Xanthomonadales, and genus of *Sphingomonas*) and TM7 (from phylum to class). There were three groups of bacteria enriched in M2, namely, Acidobacteria (the phylum and its class Acidobacteria_6, and its order iii1_15), Bacteroidetes, and Deltaproteobacteria. The Actinobacteria (from phylum to its class actinobacteria, and its orders actinomycetales and solirubrobacterales, and families of micrococcaceae and nocardioidaceae) and Firmicutes (from phylum to its class Bacilli, and to its order Bacillales) were enriched in CK (**Figure 4**).

### Bacterial Community Function Response to Microbial Inoculation

**FIGURE 5:**
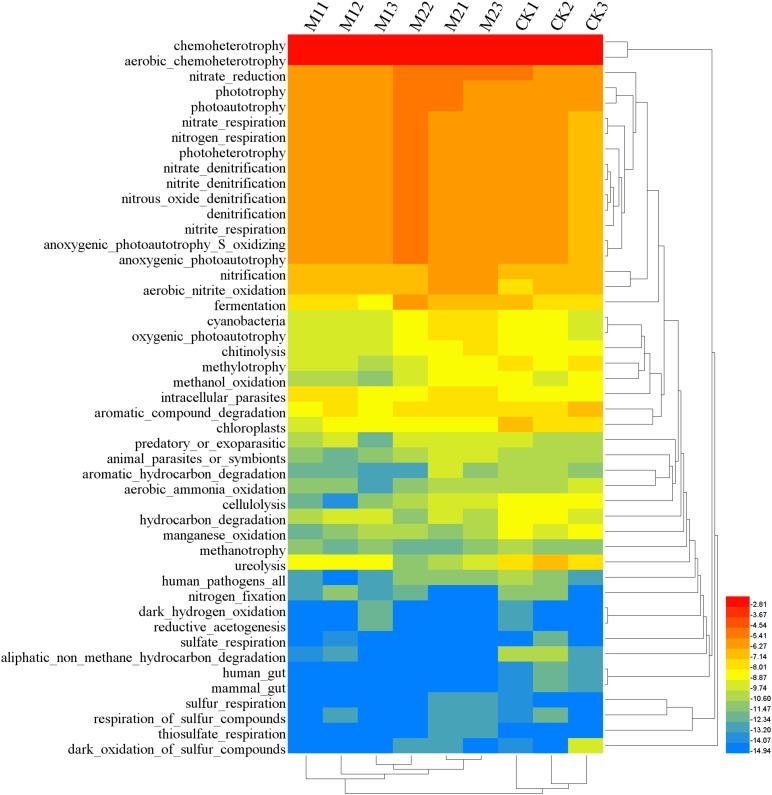
Heatmap of functional community profiles under different treatments. M1, microbial co-inoculants 1; M2, microbial co-inoculants 2; CK, non-inoculated.

FAPROTAX analysis showed that 30.26% (213 out of 704) records were assigned to at least one group. Dominant function (>1%) across all samples were chemoheterotrophy, aerobic_chemoheterotrophy, nitrate_reduction, phototrophy, photoautotrophy, nitrate_respiration, nitrogen_respiration, photoheterotrophy, anoxygenic_photoautotrophy_S_oxidizing, anoxygenic_photoautotrophy, nitrate_denitrification, nitrite_denitrification, nitrous_oxide_denitrification, denitrification, nitrite_respiration, nitrification, aerobic_nitrite_oxidation and fermentation (**Figure 5**). Microbial co-inoculation (M1 and M2) significantly increased functional groups of aerobic_nitrite_oxidation, and decreased functional groups of chloroplasts, cellulolysis, ureolysis, manganese_oxidation, human_gut, and mammal_gut (*P* < 0.05). The functional groups of intracellular_parasites were higher in M1 than in other treatments. The functional groups of phototrophy, photoautotrophy, nitrification, fermentation, cyanobacteria, oxygenic_photoautotrophy, chitinolysis and animal_parasites_or_symbionts were higher in M2 than in other treatments. The functional groups of methylotrophy, methanol_oxidation and aerobic_ammonia_oxidation were higher in CK than in other treatments (**Table 3**).

**Table 3:** Statistically significant differences in the function groups under different treatments.

	M1	M2	CK
phototrophy	1.84 ± 0.10b	2.60 ± 0.24a	1.76 ± 0.38b
photoautotrophy	1.83 ± 0.09b	2.59 ± 0.24a	1.76 ± 0.38b
nitrification	1.12 ± 0.05b	1.41 ± 0.16a	0.84 ± 0.08b
aerobic_nitrite_oxidation	1.09 ± 0.07a	1.34 ± 0.14a	0.74 ± 0.09b
fermentation	0.51 ± 0.17b	1.13 ± 0.18a	0.63 ± 0.24b
cyanobacteria	0.15 ± 0.03b	0.52 ± 0.22a	0.28 ± 0.08ab
oxygenic_photoautotrophy	0.15 ± 0.03b	0.52 ± 0.22a	0.28 ± 0.08ab
intracellular_parasites	0.43 ± 0.04a	0.36 ± 0.07ab	0.25 ± 0.02b
chitinolysis	0.19 ± 0.02b	0.38 ± 0.08a	0.31 ± 0.07ab
chloroplasts	0.23 ± 0.10b	0.30 ± 0.06b	0.68 ± 0.15a
methylotrophy	0.11 ± 0.02b	0.25 ± 0.05ab	0.35 ± 0.07a
methanol_oxidation	0.07 ± 0.02b	0.22 ± 0.05a	0.28 ± 0.07a
animal_parasites_or_symbionts	0.05 ± 0.02b	0.14 ± 0.05a	0.11 ± 0.01ab
cellulolysis	0.03 ± 0.02b	0.11 ± 0.02b	0.28 ± 0.08a
aerobic_ammonia_oxidation	0.03 ± 0.02b	0.07 ± 0.02ab	0.11 ± 0.01a
ureolysis	0.23 ± 0.02b	0.08 ± 0.04b	0.62 ± 0.11a
manganese_oxidation	0.05 ± 0.03b	0.06 ± 0.02b	0.25 ± 0.06a
human_gut	0.00 ± 0.00b	0.00 ± 0.00b	0.02 ± 0.01a
mammal_gut	0.00 ± 0.00b	0.00 ± 0.00b	0.02 ± 0.01a

*M1, microbial co-inoculants 1; M2, microbial co-inoculants 2; CK, non-inoculated. Values (means ± SD, n = 3) within the same row followed by different letters are significantly different at P < 0.05 according to Tukey’s post hoc test.*

The PCoA revealed that the bacterial community function were obviously separated by the different microbial co-inoculation treatments (Supplementary Figure S4). ANOSIM analysis showed that microbial co-inoculation treatments (global *R* = 0.6049, *P* = 0.012) were significant factors in shaping the bacterial community function. Accordingly, ADONIS analysis showed that microbial co-incubation treatment contributed 58.7% (*P* = 0.01) of community variance. Collectively, these results revealed that the microbial community function were predominantly affected by the microbial co-inoculation.

### Correlations Among Cucumber Yield, Soil Characteristics, and Specific Bacterial Taxa

Pearson’s rank correlation coefficient was used to evaluate the relationships among cucumber yield, soil characteristics and specific bacterial taxa obtained by LEfSe (Supplementary Tables S1, S2). Cucumber yield was significantly positively correlated with TN and UE. Four specific bacterial taxa of M1 (TM7-1, Legionellales, Sinobacteraceae, PRR-10) were significantly positively correlated with cucumber yield. Most of the specific bacterial taxa of M1 were significantly positively correlated with TN and IA. Some of the specific bacterial taxa of M1 were significantly positively correlated with TK, AN, UE, and significantly negatively correlated with AK, NO_3_-N, and CA (Supplementary Table S1). Ten specific bacterial taxa of M2 (Acidobacteria-6, RB25, OM190, iii1-15, Bacteroidales, N1423WL, mb2424, SJA-101, JG37-AG-70) were significantly positively correlated with cucumber yield. Most of the specific bacterial taxa of M2 were significantly positively correlated with UE and AKP. Some of the specific bacterial taxa of M2 were significantly positively correlated with APA, UE, CA, NO_3_-N, TK, AK, TN and significantly negatively correlated with AI (Supplementary Table S2).

## Discussion

Microbial co-inoculation is considered to be an innovative approach and had been applied worldwide (Baris et al., 2014; Baez-Rogelio et al., 2017). However, the underlying mechanisms of microbial co-inoculants constructions, especially the trait-based combination of distinctly different microbial species remains poorly understood. Microbial co-inoculants construction includes species selection and species integration. Most researches selected two or more compatible strains with different functions to construct co-inoculants. The common selections were nitrogen fixing bacteria (*Azospirillum* sp or Rhizobia) and biocontrol agents (*Pseudomonas* sp. or *Bacillus* sp.) (Figueiredo et al., 2008; Atieno et al., 2012b). In this study we selected three compatible strains evolving functions about nitrogen fixing, phosphate solubilizing and potassium solubilizing. The species selection was based on the consideration of three major nutrients (nitrogen, phosphorus, and potassium) in plant needs. Species integrations are essentially the ratios of microbial species in co-inoculants. The ratios of microbial species in co-inoculants were mostly 1:1 (Maheshwari et al., 2010; Yegorenkova et al., 2016). In this study, we set two ratios according to soil fertility, microbial activity and cucumber nutrient requirement. Although the influences of various factors in soil on the activity of bacteria were not considered and the formulas were not very scientific rigor, it provided a conception for rational construction of microbial co-inoculants.

The positive effects of microbial co-inoculation on plant yield, soil physiochemical properties, soil enzymes activity and soil microbial community had been extensively investigated (Atieno et al., 2012a; García de Salamone et al., 2012; Prasanna et al., 2012; Korir et al., 2017). However, multi-species, especially more than two species, with different ratios in inoculants on these factors had not been explored much. In this study, we illustrated the effect of the two ratios of multi-species in inoculants on cucumber yield, soil properties, and bacterial community in the field experiment. The ultimate goal is to choose the better construction of microbial co-inoculants.

### Effects on Colonization

Co-inoculation has been proved to increase colonization (Maheshwari et al., 2010; Figueredo et al., 2017). Our study revealed that the ratio of microbial species in inoculants did have some effects on colonization (Supplementary Figure S1). The ratio of 2:1:2 (M1) significantly increased *Flavobacterium* sp. proportions while decreased Rhizobiaceae_other and *Acinetobacter* proportions compared to CK. The ratio of 1:10:1 (M2) significantly increased *Flavobacterium* sp. and Rhizobiaceae_other proportions while decreased *Acinetobacter* proportions compared to CK. The ratio of *Flavobacterium* sp. was significant highest in M1. The ratio of *Acinetobacter* sp. was significant lowest in M2. Since *Ensifer* sp. did not detect in the high throughout sequencing results, Rhizobiaceae_other was used to represent *Ensifer* sp. NYM3. The colonization of microbial co-inoculants was not direct related to the inoculation quantity. This indicated that *Flavobacterium* sp. might have higher colonization activity while *Acinetobacter* sp. might have lower colonization activity. The colonization activity of microbial inoculants was related to strain characteristics, microbial interaction and environmental factors (van Veen et al., 1997). For more accurate evaluation of colonization, the absolute abundance of inoculated species will be future studied. *Ensifer* sp. could effectively establish the nitrogen-fixing symbiosis with leguminous crop plants (Miethling et al., 2000). The colonization activity of *Acinetobacter* sp. and *Flavobacterium* sp. was rarely reported.

### Effects on Bacterial Community Structure and Function

A few works had been focus on the effects of microbial co-inoculation on soil microbial community (Trabelsi et al., 2011). However, the effect of the ratio of microbial species in inoculation was rarely described. Our results revealed that the ratio of microbial species in inoculants had significant effects on bacterial community diversity and function. This might due to the different interactions in microbial inoculants and with indigenous bacterial community. Statistics analysis showed that Gammaproteobacteria, Acidobacteria, Nitrospirae, and Armatimonadetes were significantly increased, while Actinobacteria and Firmicutes were significantly decreased by microbial co-inoculation (M1 and M2). Both the LEfSe analysis and statistics analysis revealed that the M1 had highest Gammaproteobacteria and TM7 while The M2 had highest Bacteroidetes, Deltaproteobacteria, Gemmatimonadetes, Armatimonadetes, and Verrucomicrobia. Some works showed that Bacteroidetes might result in a healthier development for plants as animals (Perez-Jaramillo et al., 2017). This was consistence with our results that higher Bacteroidetes with higher cucumber yields. Most species of Bacteroidetes in our study were Chitinophagaceae (54∼63%) in family level. Chitinophagaceae has been proposed for their potential role in protection against soil-borne pathogens (Yin et al., 2013; Perez-Jaramillo et al., 2017). This indicated that our microbial inoculants had potential biocontrol function. Effect of inoculation with *Ensifer* sp. (*Sinorhizobium* sp.) on soil bacterial community had been deeply investigated. Field inoculation with *Sinorhizobium meliloti* L33 decreased γ-proteobacteria but increased α-proteobacteria in rhizospheres of *Medicago sativa* (Schwieger and Tebbe, 2000). Cocktail of *Ensifer* strains had no effect on the bacterial community in the rhizosphere of Acacia senegal mature trees (Herrmann et al., 2012). *Acinetobacter* sp. had been widely applied to removal of organic and metal pollution from contaminated system (Bhattacharya et al., 2014; Rojas-Tapias et al., 2014). *Flavobacterium* sp. were well known identified as phosphate solubilizer and bioinoculants by many researchers (Rathi et al., 2014). The effects of *Acinetobacter* sp. or *Flavobacterium* sp. inoculation on soil bacterial community had not been reported.

There was few report about the effect of microbial co-inoculation on soil bacterial community function. Only Babic et al. (2008) demonstrated that two indigenous *Sinorhizobium meliloti* strains inoculation affected genes abundance involved in nitrogen turnover using qPCR. The differences in bacterial diversity may result in the differences in the bacterial community function. Although the coverage of FAPROTAX was not enough, it did give some information and could be used for comparison between different treatments (Louca et al., 2016). Statistics analysis showed that functional groups of aerobic_nitrite_oxidation were higher in microbial co-inoculation (M1 and M2) treatments. This was due to the higher Nitrospirales in these microbial co-inoculation (M1 and M2) treatments. The functional groups of intracellular_parasites were higher in M1 than in other treatments. This was due to the higher Chlamydia, Rickettsiales, and Legionellales in M1. The functional groups of phototrophy, photoautotrophy, nitrification, fermentation, cyanobacteria, oxygenic_photoautotrophy, chitinolysis, and animal_parasites_or_symbionts were higher in M2 than in other treatments. This was due to the higher Cyanobacteria, Rhodoplanes, Rhodobacter, Ectothiorhodospiraceae, Nitrospirales, Nitrosovibrio, Actinomycetales, Bacteroides, Bacilli, Clostridiales, Skermanella, Erwinia, Vibrionaceae, Opitutus, Lysobacter, Prevotella, Coprococcus, Francisellaceae, Acinetobacter, Stenotrophomonas, and Chthoniobacteraceae in M2. It is need further study on how microbial inoculants mediated the bacterial community structure and function in future.

### Effects on Soil Properties

The positive effect of microbial co-inoculants on soil properties had been reported by many works. Bacteria and cyanobacteria co-inoculation could improve soil nitrogen fixing potential, organic carbon, microbial biomass carbon, and enzymes activities (Prasanna et al., 2012). Co-inoculation of *M. oryzae* CBMB20 with *A. brasilense* CW903 or *B. pyrrocinia* CBPB-HOD could increase the activity of nitrogenase, urease and phosphatase enzymes in soil (Madhaiyan et al., 2010). However, the effect of different ratios of microbial species in inoculants on soil properties was few reported. This study showed that M1 had significant higher TN, IA, and pH (*P* < 0.05), M2 had significant higher urease activity and phosphatase activity (*P* < 0.05) than other treatments. This indicated that (i) microbial co-inoculants with different ratios of microbial species both had positive effect on soil properties; (ii) the ratio of microbial species in inoculants had significant effect on soil properties. The M1 and M2 increased different properties of soil. This might due to different bacterial community structure, function and activity mediated by different microbial inoculants. The ratio of *Flavobacterium* sp. was significant higher in M1 and M2 than that in CK (Supplementary Figure S1). However, the available potassium (AK) in M1 and M2 was not significant higher than CK. This might indicate that the K-solubilizing activity of *Flavobacterium* sp. was different in soil ecological systems. Our previous work showed that *Flavobacterium* sp. KYM3 could growth on Ashby medium plates (data not shown). This indicated that *Flavobacterium* sp. KYM3 had the nitrogen fixing ability. The higher TN in microbial co-inoculation treatments (M1 and M2) might be relate with the higher ratio of *Flavobacterium* sp. in microbial co-inoculants (M1 and M2) (Supplementary Figure S1).

### Effects on Cucumber Yield

The positive effect of microbial co-inoculants on plant had been widely investigated. *Bacillus* sp. CHEP5 were reported improved *Bradyrhizobium* sp. SEMIA6144 root surface colonization and increased the yield of peanut seeds from 2.15% to 16.69% (Figueredo et al., 2017). Application of *Sinorhizobium meliloti* RMP1 and *Pseudomonas aeruginosa* GRC(2) could significantly increased biomass and yield of *B. juncea* as compared to control in field trials (Maheshwari et al., 2010). Co-inoculation of *Thiobacillus* sp. with *Rhizobium* under field condition resulted in significantly higher nodule number, nodule dry weight, plant biomass and pod yield (Anandham et al., 2007). Our study firstly revealed that co-inoculation of *Ensifer* sp. NYM3, *Acinetobacter* sp. P16 and *Flavobacterium* sp. KYM3 with different ratios under field condition could promote cucumber yields. Our results also showed that the different ratios of the microbial species in inoculants (M1 and M2) had few effect on cucumber yields. This will expand our knowledge in bacteria interaction and will guide the construction and manufacture of microbial co-inoculation or fertilizers.

### Correlation Among Cucumber Yield, Soil Properties, and Bacterial Community

**FIGURE 6:**
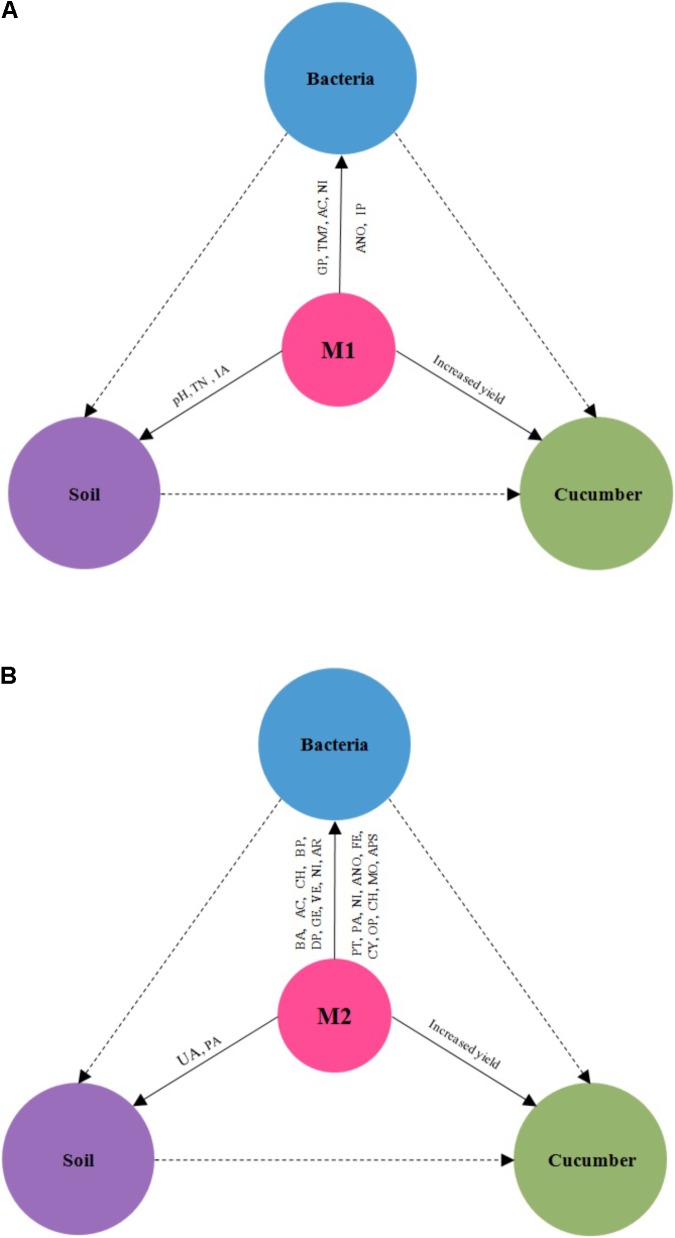
The effects of microbial co-inoculants 1 **(A)** and microbial co-inoculants 2 **(B)** on bacterial community, soil properties, and cucumber yield. M1, microbial co-inoculants 1; M2, microbial co-inoculants 2.

Microbial co-inoculants might promote cucumber yield by both the direct and indirect effects. The direct effects of microbial inoculants promote plant growth had been extensively described (Colla et al., 2015; Wang et al., 2017). Nowadays, some works had been suggested that regulation of soil bacterial community structure is one of the plant growth-promoting mechanisms of microbial inoculants (Kang et al., 2013; Rodriguez-Caballero et al., 2017). Our results did support this opinion. The results showed that M1 significantly increased TN, IA, Proteobacteria, TM7, aerobic_nitrite_oxidation and intracellular_parasites. Invertase catalyzes the hydrolysis of disaccharides to monosaccharides. Invertase plays a critical role in releasing low molecular weight sugars that are important as energy sources of microorganisms (Song et al., 2014). Cucumber yield was significantly positively correlated with TM7-1, Legionellales, Sinobacteraceae, PRR-10, TN, and UA. Most of the specific taxa of M1 was significant positive related with TN. It indicated that M1 might promote cucumber yields by mediating bacterial community structure and function about nitrogen fixing (**Figure 6A**). The results showed that M2 significantly increased Bacteroidetes, Deltaproteobacteria, Gemmatimonadetes, Armatimonadetes, Verrucomicrobia, phototrophy, photoautotrophy, nitrification, fermentation, cyanobacteria, oxygenic_photoautotrophy, chitinolysis, animal_parasites_or_symbionts, urease activity (UA) and phosphatase activity (APA). The enzyme urease catalyzes urea-N hydrolysis to NH_3_ and is produced by plants and many soil microorganisms (Fisher et al., 2017). Cucumber yield was significantly positively correlated with Acidobacteria-6, RB25, OM190, iii1-15, Bacteroidales, N1423WL, mb2424, SJA-101, JG37-AG-70, TN and UA. Most of the specific taxa of M2 was significant positive related with UA. It inferred that M2 might promote cucumber yields by mediating bacterial community structure and function about urea-N hydrolysis (**Figure 6B**). Due to the complex soil-microbe-plant ecosystem, the plant growth-promoting mechanism of microbial co-inoculants in field is still not well documented and needs to be further investigated. We will specially pay more attentions on plant nutrient contents, which could be more related to differences in soil properties as well as to bacterial community structures in future.

## Conclusion

This study described the effect of multi-species with two different ratios in inoculants on cucumber yield, soil physiochemical properties, soil enzyme activity, and bacterial community. Inoculants with different ratios of microbial species both had positive effects on cucumber yields. The ratio of microbial species in inoculants had significant effect on soil properties and bacterial community. Inoculants with different ratios of microbial species might increase cucumber yields by different ways of mediating bacterial community structure and function. This revealed that in the process of microbial co-inoculants construction, trait-based integration of different microbial species had significant effects on soil bacterial communities. It also indicated that microbial activity should be considered in the construction of microbial co-inoculants. This will expand our knowledge in bacteria interaction and plant growth promoting mechanism of bacteria and will guide microbial fertilizer formulation and application in future.

## Author Contributions

ZH, JW, and CS: conception of the study. JW, SX, QL, CS, and ZH: designed the experiments. JW, QL, WZ, SX, and YL: performed the experiments. JW and WZ: interpretation of the results. JW, CS, and ZH: wrote the manuscript.

## Conflict of Interest Statement

The authors declare that the research was conducted in the absence of any commercial or financial relationships that could be construed as a potential conflict of interest.
