# Supplementary material for: Traits-Based Integration of Multi-Species Inoculants Facilitates Shifts of Indigenous Soil Bacterial Community
Source: Front Microbiol. 2018 Jul 26;9:1692. doi: 10.3389/fmicb.2018.01692 (PMC6071577; doi:10.3389/fmicb.2018.01692)
Supplement: Supplementary file 1 [file Image_1.pdf]

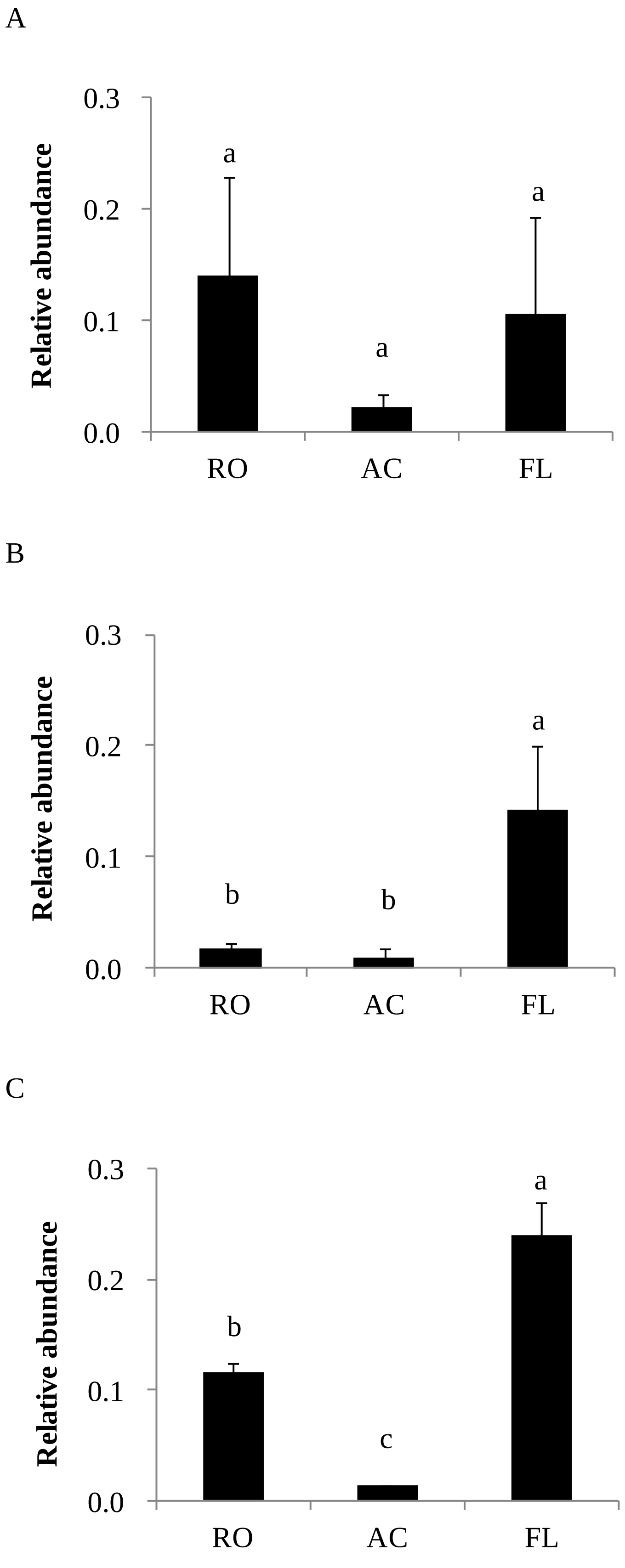

Fig. S1. Relative abundances of Rhizobiaceae_other (RA), *Acinetobacter* (AC) and

Flavobacterium (FL) under CK (A), M1 (B) and M2 (C) treatments. Different small letters indicate significant differences between groups. M1, microbial co-inoculants 1; M2, microbial co-inoculants 2; CK, non-inoculated.

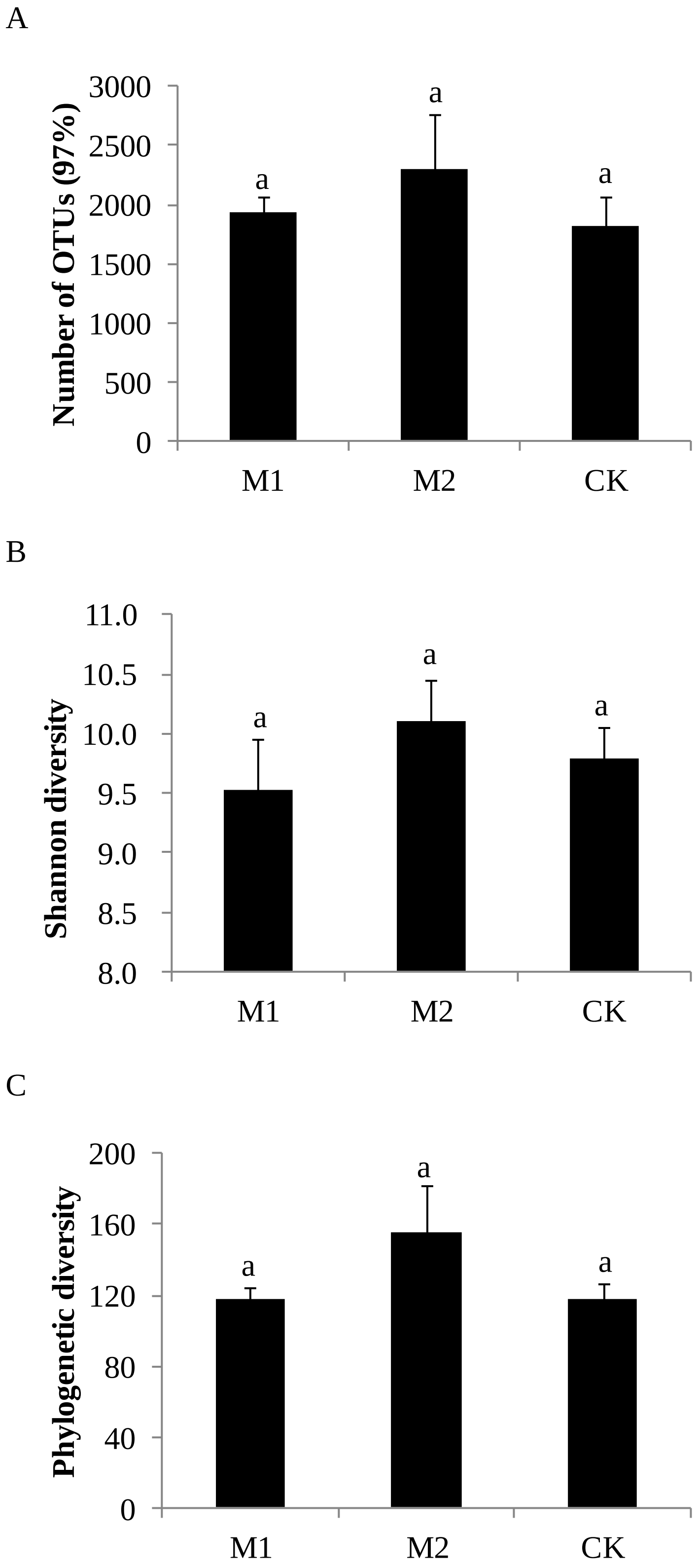

Fig. S2. The average of bacterial Phylotypes (A), Shannon diversity (B), and Phylogenetic diversity (C) under different treatments. Different small letters indicate significant differences between groups. M1, microbial co-inoculants 1; M2, microbial

co-inoculants 2; CK, non-inoculated.

A

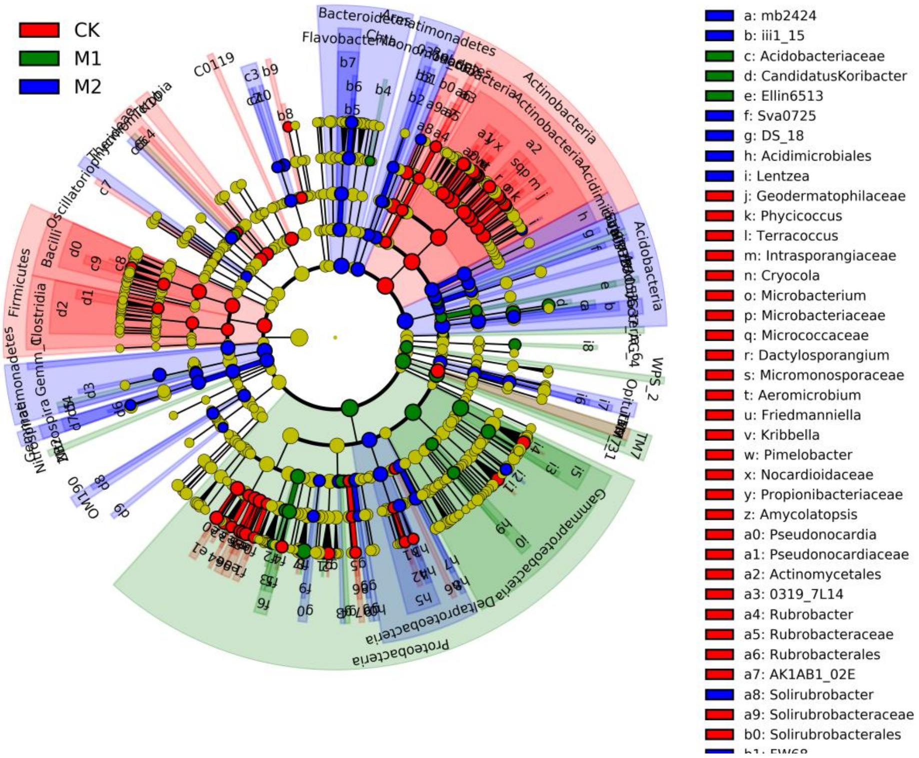
CK
M1
M2
a: mb2424
b: iii1_15
c: Acidobacteriaceae
d: CandidatusKoribacter
e: Ellin6513
f: Sva0725
g: DS_18
h: Acidimicrobiales
i: Lentzea
j: Geodermatophilaceae
k: Phycicoccus
l: Terracoccus
m: Intrasporangiaceae
n: Cryocola
o: Microbacterium
p: Microbacteriaceae
q: Micrococcaceae
r: Dactylosporangium
s: Micromonosporaceae
t: Aeromicrobium
u: Friedmanniella
v: Kribbella
w: Pimelobacter
x: Nocardioidaceae
y: Propionibacteriaceae
z: Amycolatopsis
a0: Pseudonocardia
a1: Pseudonocardiaceae
a2: Actinomycetales
a3: 0319_7L14
a4: Rubrobacter
a5: Rubrobacteraceae
a6: Rubrobacterales
a7: AK1AB1_02E
a8: Solirubrobacter
a9: Solirubrobacteraceae
b0: Solirubrobacterales
Bacteroidetes
Flavobacteriia
Armatimonadetes
Actinobacteria
Acidobacteria
Firmicutes
Bacilli
Clostridia
Gemmatimonadetes
Nitrospira
OM190
C0119
WPS_2
TM7
Gammaproteobacteria
Deltaproteobacteria
Proteobacteria

B

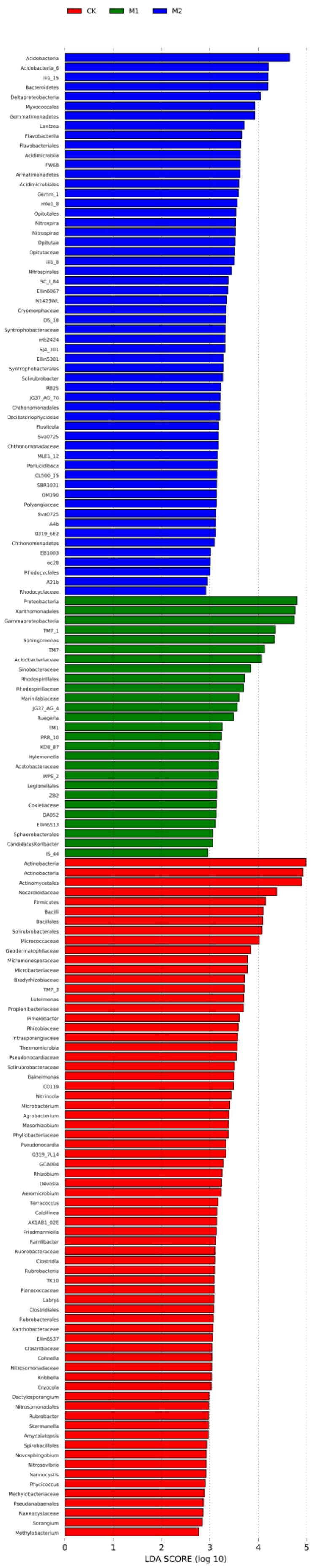

CK
M1
M2
Acidobacteria
Acidobacteria_6
iii1_15
Bacteroidetes
Deltaproteobacteria
Myxococcales
Gemmatimonadetes
Lentzea
Flavobacteriia
Flavobacteriales
Acidimicrobiia
FW68
Armatimonadetes
Acidimicrobiales
Gemm_1
mle1_8
Opitutales
Nitrospira
Nitrospirae
Opitutae
Opitutaceae
iii1_8
Nitrospirales
SC_I_84
Ellin6067
N1423WL
Cryomorphaceae
DS_18
Syntrophobacteraceae
mb2424
SJA_101
Ellin5301
Syntrophobacterales
Solirubrobacter
RB25
JG37_AG_70
Chthonomonadales
Oscillatoriophycideae
Fluviicola
Sva0725
Chthonomonadaceae
MLE1_12
Perlucidibaca
CL500_15
SBR1031
OM190
Polyangiaceae
Sva0725
A4b
0319_6E2
Chthonomonadetes
EB1003
oc28
Rhodocyclales
A21b
Rhodocyclaceae
Proteobacteria
Xanthomonadales
Gammaproteobacteria
TM7_1
Sphingomonas
TM7
Acidobacteriaceae
Sinobacteraceae
Rhodospirillales
Rhodospirillaceae
Marinilabiaceae
JG37_AG_4
Ruegeria
TM1
PRR_10
KD8_87
Hylemonella
Acetobacteraceae
WPS_2
Legionellales
ZB2
Coxiellaceae
DA052
Ellin6513
Sphaerobacterales
CandidatusKoribacter
IS_44
Actinobacteria
Actinobacteria
Actinomycetales
Nocardioidaceae
Firmicutes
Bacilli
Bacillales
Solirubrobacterales
Micrococcaceae
Geodermatophilaceae
Micromonosporaceae
Microbacteriaceae
Bradyrhizobiaceae
TM7_3
Luteimonas
Propionibacteriaceae
Pimelobacter
Rhizobiaceae
Intrasporangiaceae
Thermomicrobia
Pseudonocardiaceae
Solirubrobacteraceae
Balneimonas
C0119
Nitrincola
Microbacterium
Agrobacterium
Mesorhizobium
Phyllobacteriaceae
Pseudonocardia
0319_7L14
GCA004
Rhizobium
Devosia
Aeromicrobium
Terracoccus
Caldilinea
AK1AB1_02E
Friedmanniella
Ramlibacter
Rubrobacteraceae
Clostridia
Rubrobacteria
TK10
Planococcaceae
Labrys
Clostridiales
Rubrobacterales
Xanthobacteraceae
Ellin6537
Clostridiaceae
Cohnella
Nitrosomonadaceae
Kribbella
Cryocola
Dactylosporangium
Nitrosomonadales
Rubrobactor
Skermanella
Amycolatopsis
Spirobacillales
Novosphingobium
Nitrosovibrio
Nannocystis
Phycicoccus
Methylobacteriaceae
Pseudanabaenales
Nannocystaceae
Sorangium
Methylobacterium
0
1
2
3
4
5
LDA SCORE (log 10)

Fig. S3. Cladogram (A) and LDA score (B) of LEfSe analysis of bacterial community among CK (Red), M1 (green) and M2 (blue) treatments. The taxa with meeting a significant LDA threshold value of > 2 were shown. M1, microbial co-inoculants 1; M2, microbial co-inoculants 2; CK, non-inoculated.

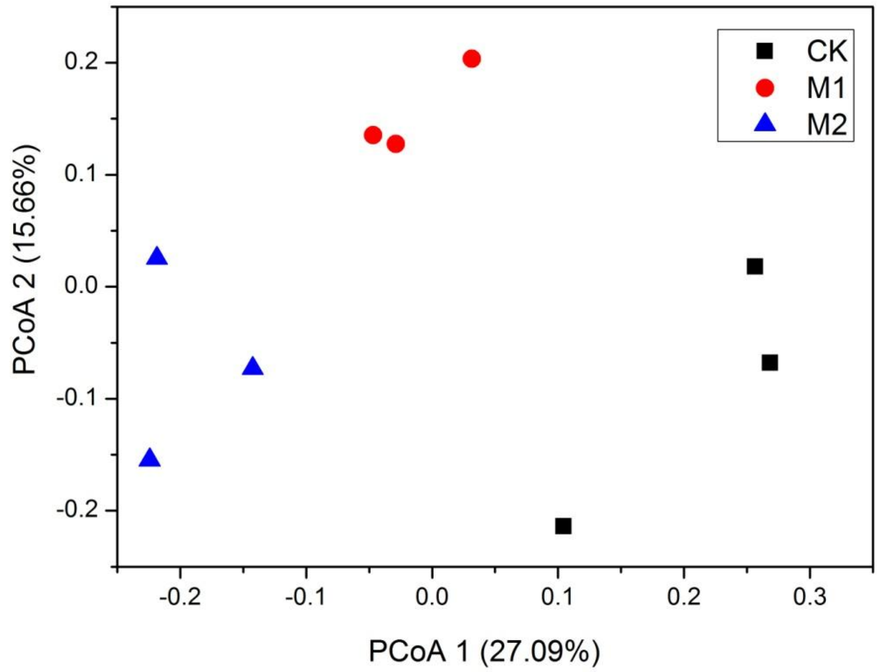

Fig. S4. Principal coordinates analysis (PCoA) based on Gower dissimilarities in terms of functional groups under different treatments. M1, microbial co-inoculants 1; M2, microbial co-inoculants 2; CK, non-inoculated.
