# Supplementary material for: Traits-Based Integration of Multi-Species Inoculants Facilitates Shifts of Indigenous Soil Bacterial Community
Source: Front Microbiol. 2018 Jul 26;9:1692. doi: 10.3389/fmicb.2018.01692 (PMC6071577; doi:10.3389/fmicb.2018.01692)
Supplement: Supplementary file 2 [file Table_1.DOCX]

**Table S1**

Correlation coefficient among specific bacterial taxa of microbial co-inoculants 1 (M1), soil properties and cucumber yield.

|  | **Yield** | **OM** | **TN** | **TP** | **TK** | **AN** | **AP** | **AK** | **NO_3_-N** | **IA** | **UA** | **APA** | **CAT** |
| --- | --- | --- | --- | --- | --- | --- | --- | --- | --- | --- | --- | --- | --- |
| Yield | 1 | 0.034 | **0.670*** | -0.214 | 0.633 | 0.454 | 0.062 | -0.316 | 0.123 | 0.434 | **0.741*** | 0.228 | -0.391 |
| Bacteria\|Proteobacteria | 0.482 | -0.259 | **0.930^**^** | 0.084 | 0.646 | 0.577 | 0.351 | -0.567 | -0.351 | **0.819^**^** | 0.523 | -0.148 | -0.529 |
| Bacteria\|TM7 | 0.659 | -0.318 | **0.925^**^** | -0.179 | **0.672^*^** | 0.564 | 0.359 | **-0.815^**^** | -0.516 | **0.856^**^** | 0.470 | -0.358 | **-0.692^*^** |
| Bacteria\|WPS-2 | 0.571 | -0.361 | **0.899^**^** | -0.088 | **0.731^*^** | 0.504 | 0.260 | **-0.764^*^** | -0.514 | **0.876^**^** | 0.458 | -0.344 | **-0.766^*^** |
| Bacteria\|AD3\|JG37-AG-4 | 0.466 | -0.367 | **0.828^**^** | -0.117 | 0.658 | 0.644 | 0.509 | **-0.716^*^** | -0.663 | **0.967^**^** | 0.193 | -0.570 | **-0.856^**^** |
| Bacteria\|Acidobacteria\|DA052 | 0.516 | -0.363 | **0.871^**^** | -0.227 | 0.566 | 0.529 | 0.502 | **-0.670^*^** | -0.507 | **0.900^**^** | 0.352 | -0.394 | -0.655 |
| Bacteria\|Acidobacteria\|TM1 | 0.459 | -0.483 | **0.821^**^** | -0.183 | 0.618 | 0.539 | 0.361 | **-0.805^**^** | -0.647 | **0.955^**^** | 0.236 | -0.575 | **-0.831^**^** |
| Bacteria\|OD1\|ZB2 | 0.193 | -0.529 | 0.626 | -0.196 | 0.543 | 0.486 | 0.452 | -0.552 | **-0.667^*^** | **0.927^**^** | 0.008 | -0.629 | **-0.785^*^** |
| Bacteria\|TM7\|TM7-1 | **0.740^*^** | -0.248 | **0.966^**^** | -0.154 | **0.728^*^** | 0.564 | 0.333 | **-0.740^*^** | -0.404 | **0.818^**^** | 0.604 | -0.201 | -0.630 |
| Bacteria\|Acidobacteria\|DA052\|Ellin6513 | 0.516 | -0.363 | **0.871^**^** | -0.227 | 0.566 | 0.529 | 0.502 | **-0.670^*^** | -0.507 | **0.900^**^** | 0.352 | -0.394 | -0.655 |
| Bacteria\|Chloroflexi\|Thermomicrobia\|Sphaerobacterales | 0.158 | -0.384 | 0.651 | -0.147 | 0.417 | 0.435 | 0.586 | **-0.758^*^** | **-0.830^**^** | **0.880^**^** | -0.104 | **-0.780^*^** | **-0.834^**^** |
| Bacteria\|Gemmatimonadetes\|Gemmatimonadetes\|KD8-87 | 0.601 | -0.483 | **0.767^*^** | -0.414 | **0.728^*^** | 0.413 | 0.256 | -0.591 | -0.425 | **0.839^**^** | 0.459 | -0.288 | -0.662 |
| Bacteria\|Proteobacteria\|Alphaproteobacteria\|Rhodospirillales | 0.653 | -0.285 | **0.945^**^** | -0.063 | **0.818^**^** | **0.671^*^** | 0.377 | -0.625 | -0.442 | **0.899^**^** | 0.527 | -0.237 | **-0.706^*^** |
| Bacteria\|Proteobacteria\|Betaproteobacteria\|IS-44 | 0.501 | 0.151 | **0.926^**^** | 0.277 | 0.665 | 0.633 | 0.560 | -0.579 | -0.442 | 0.636 | 0.484 | -0.103 | -0.493 |
| Bacteria\|Proteobacteria\|Gammaproteobacteria\|Legionellales | **0.767^*^** | -0.161 | **0.861^**^** | -0.080 | **0.833^**^** | 0.536 | 0.132 | -0.317 | -0.021 | 0.612 | **0.851^**^** | 0.266 | -0.315 |
| Bacteria\|Proteobacteria\|Gammaproteobacteria\|Xanthomonadales | 0.535 | -0.327 | **0.923^**^** | -0.012 | **0.688^*^** | 0.660 | 0.421 | **-.762^*^** | -0.581 | **0.941^**^** | 0.350 | -0.443 | **-0.788^*^** |
| Bacteria\|Acidobacteria\|Acidobacteriia\|Acidobacteriales\|Acidobacteriaceae | 0.447 | -0.399 | **0.849^**^** | -0.152 | 0.611 | 0.560 | 0.477 | **-0.790^*^** | **-0.675^*^** | **0.952^**^** | 0.222 | -0.573 | **-0.823^**^** |
| Bacteria\|Bacteroidetes\|Bacteroidia\|Bacteroidales\|Marinilabiaceae | 0.179 | -0.650 | 0.655 | -0.220 | 0.452 | 0.411 | 0.322 | **-0.685^*^** | -0.653 | **0.936^**^** | 0.045 | -0.658 | **-0.747^*^** |
| Bacteria\|Proteobacteria\|Alphaproteobacteria\|Rhodospirillales\|Acetobacteraceae | -0.171 | -0.349 | 0.346 | -0.151 | 0.158 | 0.309 | 0.665 | -0.480 | **-0.793^*^** | **0.705^*^** | -0.407 | **-0.818^**^** | -0.646 |
| Bacteria\|Proteobacteria\|Gammaproteobacteria\|Legionellales\|Coxiellaceae | 0.585 | -0.285 | **0.935^**^** | -0.013 | **0.801^**^** | 0.557 | 0.261 | -0.514 | -0.296 | **0.800^**^** | 0.658 | -0.032 | -0.518 |
| Bacteria\|Proteobacteria\|Gammaproteobacteria\|Xanthomonadales\|Sinobacteraceae | **0.682^*^** | -0.184 | **0.979^**^** | 0.123 | **0.794^*^** | **0.666^*^** | 0.244 | **-0.687^*^** | -0.367 | **0.803^**^** | 0.619 | -0.136 | -0.641 |
| Bacteria\|WS3\|PRR-12\|Sediment-1\|PRR-10 | **0.751^*^** | -0.140 | **0.938^**^** | -0.029 | **0.758^*^** | 0.555 | 0.225 | -0.451 | -0.109 | **0.673^*^** | **0.801^**^** | 0.157 | -0.378 |
| Bacteria\|Acidobacteria\|Acidobacteriia\|Acidobacteriales\|Koribacteraceae\|Candidatus Koribacter | 0.497 | -0.130 | **0.827^**^** | 0.131 | **0.780^*^** | 0.546 | 0.230 | -0.234 | -0.106 | 0.615 | **0.705^*^** | 0.218 | -0.283 |
| Bacteria\|Proteobacteria\|Alphaproteobacteria\|Rhodobacterales\|Rhodobacteraceae\|Ruegeria | 0.331 | -0.538 | 0.631 | -0.375 | 0.589 | 0.244 | 0.237 | -0.631 | -0.565 | **0.796^*^** | 0.232 | -0.459 | **-0.701^*^** |
| Bacteria\|Proteobacteria\|Betaproteobacteria\|Burkholderiales\|Comamonadaceae\|Hylemonella | 0.362 | -0.349 | **0.801^**^** | -0.078 | 0.507 | 0.604 | 0.561 | **-0.675^*^** | -0.625 | **0.928^**^** | 0.140 | -0.561 | **-0.746^*^** |
| Bacteria\|Proteobacteria\|Gammaproteobacteria | 0.590 | -0.290 | **0.950^**^** | -0.019 | **0.742^*^** | 0.641 | 0.399 | **-0.730^*^** | -0.528 | **0.919^**^** | 0.439 | -0.352 | **-0.766^*^** |

OM, organic matter; TN, total nitrogen; TP, total phosphate; TK, total potassium; AN, available nitrogen; AP, available phosphate; AK, available potassium; NO_3_-N, nitrate nitrogen; IA, invertase activity; UA, urease activity; APA, alkaline phosphatase activity; CA, catalase activity. Values in bold indicate statistically significant, **P* < 0.05, ***P* < 0.01.
