# Supplementary material for: Traits-Based Integration of Multi-Species Inoculants Facilitates Shifts of Indigenous Soil Bacterial Community
Source: Front Microbiol. 2018 Jul 26;9:1692. doi: 10.3389/fmicb.2018.01692 (PMC6071577; doi:10.3389/fmicb.2018.01692)
Supplement: Supplementary file 3 [file Table_2.DOCX]

**Table S2**

Correlation coefficient among specific bacterial taxa of microbial co-inoculants 2 (M2), soil properties and cucumber yield.

|  | **Yield** | **OM** | **TN** | **TP** | **TK** | **AN** | **AP** | **AK** | **NO_3_-N** | **IA** | **UA** | **APA** | **CAT** |
| --- | --- | --- | --- | --- | --- | --- | --- | --- | --- | --- | --- | --- | --- |
| Yield | 1 | 0.034 | **0.670*** | -0.214 | 0.633 | 0.454 | 0.062 | -0.316 | 0.123 | 0.434 | **0.741*** | 0.228 | -0.391 |
| Bacteria\|Acidobacteria | **0.768^*^** | 0.043 | **0.754^*^** | 0.025 | **0.688^*^** | 0.329 | -0.063 | -0.213 | 0.226 | 0.315 | **0.973^**^** | 0.542 | -0.070 |
| Bacteria\|Armatimonadetes | 0.399 | 0.304 | 0.152 | 0.161 | 0.290 | -0.047 | -0.410 | 0.332 | 0.657 | -0.369 | **0.813^**^** | **0.940^**^** | 0.546 |
| Bacteria\|Gemmatimonadetes | 0.465 | 0.350 | 0.126 | 0.166 | 0.397 | 0.058 | -0.362 | 0.420 | 0.651 | -0.331 | **0.777^*^** | **0.926^**^** | 0.440 |
| Bacteria\|Nitrospirae | 0.631 | -0.016 | 0.469 | -0.146 | 0.339 | 0.020 | -0.290 | 0.014 | 0.538 | 0.018 | **0.952^**^** | **0.741^*^** | 0.325 |
| Bacteria\|Acidobacteria\|Acidobacteria-6 | **0.698^*^** | 0.111 | 0.619 | 0.015 | 0.549 | 0.168 | -0.149 | -0.069 | 0.382 | 0.138 | **0.973^**^** | **0.681^*^** | 0.104 |
| Bacteria\|Acidobacteria\|RB25 | **0.703^*^** | 0.096 | 0.529 | -0.099 | 0.465 | 0.156 | -0.149 | 0.025 | 0.468 | 0.059 | **0.958^**^** | **0.731^*^** | 0.249 |
| Bacteria\|Acidobacteria\|Sva0725 | 0.461 | 0.365 | 0.378 | 0.392 | 0.368 | 0.159 | -0.288 | 0.096 | 0.490 | -0.194 | **0.822^**^** | **0.800^**^** | 0.349 |
| Bacteria\|Acidobacteria\|iii1-8 | 0.545 | 0.354 | 0.512 | 0.403 | 0.505 | 0.247 | -0.197 | -0.030 | 0.356 | -0.038 | **0.839^**^** | **0.692^*^** | 0.142 |
| Bacteria\|Actinobacteria\|Acidimicrobiia | -0.066 | 0.433 | -0.552 | 0.149 | -0.193 | -0.327 | -0.490 | **0.832^**^** | **0.812^**^** | **-0.805^**^** | 0.219 | **0.866^**^** | **0.777^*^** |
| Bacteria\|Armatimonadetes\|0319-6E2 | -0.051 | 0.415 | -0.244 | 0.340 | -0.048 | -0.320 | -0.506 | 0.553 | **0.687^*^** | **-0.675^*^** | 0.444 | **0.896^**^** | **0.714^*^** |
| Bacteria\|Bacteroidetes\|Flavobacteriia | -0.158 | 0.555 | -0.409 | 0.463 | -0.047 | -0.222 | -0.408 | **0.677^*^** | 0.598 | **-0.735^*^** | 0.199 | **0.781^*^** | 0.622 |
| Bacteria\|Cyanobacteria\|Oscillatoriophycideae | 0.216 | 0.195 | -0.257 | -0.036 | -0.041 | -0.261 | -0.560 | **0.686^*^** | **0.907^**^** | -0.563 | 0.559 | **0.964^**^** | **0.733^*^** |
| Bacteria\|Gemmatimonadetes\|Gemm-1 | 0.644 | 0.190 | 0.508 | 0.203 | 0.526 | 0.218 | -0.293 | 0.033 | 0.479 | 0.007 | **0.942^**^** | **0.765^*^** | 0.189 |
| Bacteria\|Planctomycetes\|OM190 | **0.699^*^** | 0.207 | 0.389 | -0.038 | 0.490 | 0.121 | -0.251 | 0.175 | 0.562 | -0.079 | **0.933^**^** | **0.827^**^** | 0.271 |
| Bacteria\|Proteobacteria\|Deltaproteobacteria | -0.011 | 0.382 | -0.357 | 0.308 | -0.081 | -0.269 | -0.604 | 0.653 | **0.789^*^** | **-0.720^*^** | 0.400 | **0.913^**^** | **0.742^*^** |
| Bacteria\|Verrucomicrobia\|Opitutae | 0.510 | 0.382 | 0.110 | 0.162 | 0.221 | 0.023 | -0.374 | 0.411 | **0.758^*^** | -0.365 | **0.762^*^** | **0.936^**^** | 0.485 |
| Bacteria\|Acidobacteria\|Acidobacteria-6\|iii1-15 | **0.696^*^** | 0.123 | 0.601 | 0.006 | 0.544 | 0.154 | -0.155 | -0.051 | 0.395 | 0.116 | **0.973^**^** | **0.696^*^** | 0.122 |
| Bacteria\|Acidobacteria\|Sva0725\|Sva0725 | 0.461 | 0.365 | 0.378 | 0.392 | 0.368 | 0.159 | -0.288 | 0.096 | 0.490 | -0.194 | **0.822^**^** | **0.800^**^** | 0.349 |
| Bacteria\|Acidobacteria\|iii1-8\|DS-18 | 0.451 | 0.384 | 0.406 | 0.405 | 0.375 | 0.120 | -0.262 | 0.025 | 0.423 | -0.170 | **0.802^**^** | **0.745^*^** | 0.272 |
| Bacteria\|Actinobacteria\|Acidimicrobiia\|Acidimicrobiales | -0.066 | 0.433 | -0.552 | 0.149 | -0.193 | -0.327 | -0.490 | **0.832^**^** | **0.812^**^** | **-0.805^**^** | 0.219 | **0.866^**^** | 0.777^*^ |
| Bacteria\|Armatimonadetes\|Armatimonadia\|FW68 | 0.274 | 0.268 | 0.118 | 0.006 | 0.288 | -0.188 | -0.316 | 0.242 | 0.472 | -0.404 | **0.743^*^** | **0.841^**^** | 0.577 |
| Bacteria\|Armatimonadetes\|Chthonomonadetes\|Chthonomonadales | 0.559 | 0.208 | 0.223 | 0.076 | 0.311 | -0.019 | -0.476 | 0.245 | **0.695^*^** | -0.255 | **0.871^**^** | **0.895^**^** | 0.413 |
| Bacteria\|Bacteroidetes\|Bacteroidia\|Bacteroidales | **0.777^*^** | -0.087 | 0.251 | -0.290 | 0.312 | 0.254 | -0.281 | 0.139 | 0.553 | 0.103 | 0.664 | 0.501 | 0.082 |
| Bacteria\|Bacteroidetes\|Flavobacteriia\|Flavobacteriales | -0.158 | 0.553 | -0.407 | 0.467 | -0.044 | -0.216 | -0.410 | **0.680^*^** | 0.601 | **-0.732^*^** | 0.202 | **0.784^*^** | 0.623 |
| Bacteria\|Chloroflexi\|Anaerolineae\|SBR1031 | 0.557 | 0.242 | 0.414 | -0.081 | 0.190 | -0.111 | -0.130 | 0.024 | 0.508 | -0.129 | **0.857^**^** | **0.745^*^** | 0.370 |
| Bacteria\|Cyanobacteria\|4C0d-2\|MLE1-12 | 0.311 | 0.398 | 0.076 | 0.122 | 0.172 | -0.113 | -0.285 | 0.411 | 0.660 | -0.466 | **0.735^*^** | **0.955^**^** | 0.667^*^ |
| Bacteria\|Gemmatimonadetes\|Gemmatimonadetes\|N1423WL | **0.731^*^** | 0.067 | 0.605 | 0.092 | **0.692^*^** | 0.402 | -0.171 | 0.002 | 0.368 | 0.197 | **0.949^**^** | 0.662 | 0.065 |
| Bacteria\|Nitrospirae\|Nitrospira\|Nitrospirales | 0.631 | -0.016 | 0.469 | -0.146 | 0.339 | 0.020 | -0.290 | 0.014 | 0.538 | 0.018 | **0.952^**^** | **0.741^*^** | 0.325 |
| Bacteria\|Planctomycetes\|OM190\|CL500-15 | 0.349 | 0.419 | 0.233 | 0.429 | 0.277 | 0.041 | -0.340 | 0.235 | 0.563 | -0.304 | **0.732^*^** | **0.841^**^** | 0.394 |
| Bacteria\|Planctomycetes\|Phycisphaerae\|mle1-8 | 0.341 | 0.180 | 0.158 | 0.067 | 0.299 | -0.202 | -0.470 | 0.220 | 0.557 | -0.308 | **0.784^*^** | **0.837^**^** | 0.429 |
| Bacteria\|Proteobacteria\|Betaproteobacteria\|A21b | 0.574 | -0.092 | 0.443 | -0.087 | 0.550 | 0.139 | -0.350 | 0.036 | 0.422 | 0.060 | **0.924^**^** | **0.698^*^** | 0.255 |
| Bacteria\|Proteobacteria\|Betaproteobacteria\|Ellin6067 | 0.471 | -0.028 | 0.438 | -0.135 | 0.381 | -0.007 | -0.204 | 0.109 | 0.471 | 0.009 | **0.908^**^** | **0.754^*^** | 0.390 |
| Bacteria\|Proteobacteria\|Betaproteobacteria\|Rhodocyclales | 0.490 | 0.099 | 0.177 | -0.271 | 0.167 | -0.108 | -0.222 | 0.326 | 0.658 | -0.215 | **0.782^*^** | **0.825^**^** | 0.542 |
| Bacteria\|Proteobacteria\|Betaproteobacteria\|SC-I-84 | 0.550 | 0.275 | 0.471 | 0.132 | 0.532 | 0.091 | -0.183 | 0.040 | 0.382 | -0.082 | **0.914^**^** | **0.773^*^** | 0.255 |
| Bacteria\|Proteobacteria\|Deltaproteobacteria\|Myxococcales | -0.063 | 0.381 | -0.433 | 0.328 | -0.182 | -0.302 | -0.641 | 0.662 | **0.806^**^** | **-0.764^*^** | 0.315 | **0.872^**^** | **0.755^*^** |
| Bacteria\|Proteobacteria\|Deltaproteobacteria\|Syntrophobacterales | 0.546 | 0.245 | 0.604 | 0.336 | 0.602 | 0.300 | -0.126 | -0.036 | 0.303 | 0.088 | **0.890^**^** | **0.675^*^** | 0.114 |
| Bacteria\|Verrucomicrobia\|Opitutae\|Opitutales | 0.542 | 0.348 | 0.140 | 0.170 | 0.234 | 0.055 | -0.396 | 0.379 | **0.760^*^** | -0.322 | **0.775^*^** | **0.916^**^** | 0.445 |
| Bacteria\|Acidobacteria\|Acidobacteria-6\|iii1-15\|mb2424 | **0.726^*^** | 0.077 | 0.365 | -0.240 | 0.340 | -0.026 | -0.294 | 0.122 | 0.614 | -0.056 | **0.924^**^** | **0.779^*^** | 0.283 |
| Bacteria\|Armatimonadetes\|Chthonomonadetes\|Chthonomonadales\|Chthonomonadaceae | 0.559 | 0.208 | 0.223 | 0.076 | 0.311 | -0.019 | -0.476 | 0.245 | **0.695^*^** | -0.255 | **0.871^**^** | **0.895^**^** | 0.413 |
| Bacteria\|Bacteroidetes\|Flavobacteriia\|Flavobacteriales\|Cryomorphaceae | 0.032 | 0.508 | -0.226 | 0.298 | -0.106 | -0.357 | -0.445 | 0.499 | **0.692^*^** | **-0.699^*^** | 0.445 | **0.881^**^** | **0.683^*^** |
| Bacteria\|Chloroflexi\|Anaerolineae\|SBR1031\|A4b | 0.571 | 0.212 | 0.442 | -0.018 | 0.288 | 0.023 | -0.112 | 0.118 | 0.534 | -0.053 | **0.874^**^** | **0.773^*^** | 0.337 |
| Bacteria\|Chloroflexi\|Anaerolineae\|SBR1031\|SJA-101 | **0.746^*^** | 0.101 | **0.730^*^** | 0.044 | 0.581 | 0.357 | 0.009 | -0.168 | 0.271 | 0.269 | **0.938^**^** | 0.560 | 0.029 |
| Bacteria\|Chloroflexi\|Anaerolineae\|SBR1031\|oc28 | 0.456 | 0.170 | 0.416 | -0.069 | 0.340 | -0.193 | -0.202 | -0.060 | 0.347 | -0.105 | **0.846^**^** | **0.680^*^** | 0.267 |
| Bacteria\|Gemmatimonadetes\|Gemmatimonadetes\|Gemmatimonadales\|Ellin5301 | -0.034 | 0.421 | -0.372 | 0.133 | -0.138 | -0.426 | -0.499 | 0.612 | **0.730^*^** | **-0.790^*^** | 0.403 | **0.911^**^** | **0.821^**^** |
| Bacteria\|Proteobacteria\|Betaproteobacteria\|Rhodocyclales\|Rhodocyclaceae | 0.490 | 0.099 | 0.177 | -0.271 | 0.167 | -0.108 | -0.222 | 0.326 | 0.658 | -0.215 | **0.782^*^** | **0.825^**^** | 0.542 |
| Bacteria\|Proteobacteria\|Deltaproteobacteria\|Myxococcales\|Polyangiaceae | -0.257 | 0.385 | **-0.697^*^** | 0.093 | -0.480 | -0.559 | -0.548 | **0.776^*^** | **0.800^**^** | **-0.923^**^** | 0.057 | **0.769^*^** | **0.878^**^** |
| Bacteria\|Proteobacteria\|Deltaproteobacteria\|Syntrophobacterales\|Syntrophobacteraceae | 0.546 | 0.245 | 0.604 | 0.336 | 0.602 | 0.300 | -0.126 | -0.036 | 0.303 | 0.088 | **0.890^**^** | **0.675^*^** | 0.114 |
| Bacteria\|Verrucomicrobia\|Opitutae\|Opitutales\|Opitutaceae | 0.542 | 0.348 | 0.140 | 0.170 | 0.234 | 0.055 | -0.396 | 0.379 | **0.760^*^** | -0.322 | **0.775^*^** | **0.916^**^** | 0.445 |
| Bacteria\|Actinobacteria\|Actinobacteria\|Actinomycetales\|Actinosynnemataceae\|Lentzea | 0.270 | 0.331 | -0.120 | 0.150 | 0.046 | -0.204 | -0.513 | 0.586 | **0.853^**^** | -0.524 | 0.631 | **0.980^**^** | 0.629 |
| Bacteria\|Actinobacteria\|Thermoleophilia\|Solirubrobacterales\|Solirubrobacteraceae\|Solirubrobacter | 0.475 | 0.188 | -0.003 | -0.186 | 0.136 | -0.141 | -0.408 | 0.416 | **0.750^*^** | -0.398 | **0.726^*^** | **0.888^**^** | 0.594 |
| Bacteria\|Bacteroidetes\|Flavobacteriia\|Flavobacteriales\|Cryomorphaceae\|Fluviicola | 0.239 | 0.411 | -0.175 | 0.076 | -0.099 | -0.344 | -0.476 | 0.516 | **0.828^**^** | -0.640 | 0.583 | **0.954^**^** | **0.708^*^** |
| Bacteria\|Nitrospirae\|Nitrospira\|Nitrospirales\|Nitrospiraceae\|JG37-AG-70 | **0.743^*^** | -0.034 | 0.309 | -0.228 | 0.354 | 0.103 | -0.392 | 0.147 | 0.639 | -0.015 | **0.867^**^** | **0.723^*^** | 0.257 |
| Bacteria\|Proteobacteria\|Gammaproteobacteria\|Pseudomonadales\|Moraxellaceae\|Perlucidibaca | 0.116 | 0.423 | 0.076 | 0.479 | 0.120 | -0.124 | -0.378 | 0.288 | 0.534 | -0.415 | 0.564 | **0.787^*^** | 0.456 |

OM, organic matter; TN, total nitrogen; TP, total phosphate; TK, total potassium; AN, available nitrogen; AP, available phosphate; AK, available potassium; NO_3_-N, nitrate nitrogen; IA, invertase activity; UA, urease activity; APA, alkaline phosphatase activity; CA, catalase activity. Values in bold indicate statistically significant, **P* < 0.05, ***P* < 0.01.
